# Molecular Modeling of Lectin-Like Protein from *Acacia farnesiana* Reveals a Possible Anti-Inflammatory Mechanism in Carrageenan-Induced Inflammation

**DOI:** 10.1155/2013/253483

**Published:** 2013-12-30

**Authors:** Vanessa Erika Ferreira Abrantes, Bruno Anderson Matias da Rocha, Raphael Batista da Nóbrega, José Caetano Silva-Filho, Claudener Souza Teixeira, Benildo Sousa Cavada, Carlos Alberto de Almeida Gadelha, Sergio Henrique Ferreira, Jozi Godoy Figueiredo, Tatiane Santi-Gadelha, Plinio Delatorre

**Affiliations:** ^1^Departamento de Biologia Molecular, Universidade Federal da Paraíba, Campus I, Cidade Universitária, 58059-900 João Pessoa, PB, Brazil; ^2^Departamento de Bioquímica e Biologia Molecular, Universidade Federal do Ceará, BioMol-Lab, Campus do Pici S/N, 60440-970 Fortaleza, CE, Brazil; ^3^Departamento de Farmacologia, Faculdade de Medicina de Ribeirão Preto, Universidade de São Paulo, 14049-900 Ribeirão Preto, SP, Brazil

## Abstract

*Acacia farnesiana* lectin-like protein (AFAL) is a chitin-binding protein and has been classified as phytohaemagglutinin from *Phaseolus vulgaris* (PHA). Legume lectins are examples for structural studies, and this family of proteins shows a remarkable conservation in primary, secondary, and tertiary structures. Lectins have ability to reduce the effects of inflammation caused by phlogistic agents, such as carrageenan (CGN). This paper explains the anti-inflammatory activity of AFAL through structural comparison with anti-inflammatory legume lectins. The AFAL model was obtained by molecular modeling and molecular docking with glycan and carrageenan were performed to explain the AFAL structural behavior and biological activity. *Pisum sativum* lectin was the best template for molecular modeling. The AFAL structure model is folded as a **β** sandwich. The model differs from template in loop regions, number of **β** strands and carbohydrate-binding site. Carrageenan and glycan bind to different sites on AFAL. The ability of AFAL binding to carrageenan can be explained by absence of the sixth **β**-strand (posterior **β** sheets) and two **β** strands in frontal region. AFAL can inhibit pathway inflammatory process by carrageenan injection by connecting to it and preventing its entry into the cell and triggers the reaction.

## 1. Introduction

*Acacia farnesiana* is a member of Leguminosae family, and it is included in the Mimosoideae subfamily. *Acacia farnesiana* lectin-like protein (AFAL) has been purified and classified as PHA-like lectin [1]. Lectins are defined as carbohydrate-binding glyco/proteins that are able to agglutinate cells, without enzymatic activity toward carbohydrate or glycoconjugates [2–4]. Plant lectins have been structural models for studying carbohydrate recognition and oligomerization states [5–7]. AFAL has a time-dependent oligomerization and it is characterized as a chitin-binding lectin based on its affinity by glycans, which elect it as a good example for structural studies. AFAL hemagglutinating activity was not inhibited by carbohydrate compounds neither the methylated or (GlcNAc) derivative ones [1]. However, *Phaseolus vulgaris* isolectins (PHA) usually bind to 1,6-branched GlcNAc containing *N*-glycans and the inhibition test result is normally positive for this inhibitor [8–10].

Legume lectins have a high degree of sequence similarity and important functional regions with amino acid residue conserved [11]. Legume lectins despite the structural identity exhibit considerable differences in biological activities and carbohydrate recognition [12]. Comparisons of these sequences and structures have established that differences in carbohydrate specificity appear to be primarily due to differences in amino acid residues residing in adjacent loops to the carbohydrate-binding site [13–16]. The conformation of these loops is determined by the presence of calcium and transition metal ions coordinated in the protein structure [17–19]. One of the most studied biological activities of lectins is the ability to reduce the effects of inflammation caused by certain phlogistic agents, such as carrageenan (CGN) [20]. Many molecules with this ability were considered for their potential for the development of drugs with anti-inflammatory action [21]. CGN is a sulfated polysaccharide obtained from several species of red algae. It is frequently incorporated into processed foods to improve texture and solubility [22]. CGN exposure possibly causes inflammation by inducing an increase in interleukin-8 (IL-8) secretion in cells. This increase was demonstrated in tissue cultures, animal colon, and human tissues [23, 24]. Mechanisms of CGN-induced IL-8 activation require nuclear localization of nuclear factor *κ*B (NF*κ*B) and can proceed through a toll-like-receptor-4- (TLR4-) B-cell-lymphoma/leukemia-10- (Bcl10-) mediated pathway [25].

The aim of this paper is to obtain a structural model of AFAL by molecular modeling to explain AFAL structural behavior and biological activity based on structure/function analysis. The analysis was made using molecular docking with oligosaccharides and CGN and investigating the anti-inflammatory activity of AFAL in model carrageenan-induced inflammation.

## 2. Material and Methods

### 2.1. Protein Purification and Haemagglutinating Assays

Purified AFAL protein was obtained from seeds according to established protocols by Santi-Gadelha and coworkers [1]. Previous studies showed that AFAL presents only hemagglutinating activity using rabbit erythrocytes. Haemagglutinating activity (HA) and inhibition tests were carried out according to standard procedure [9]. The negative control was composed of 100 *μ*L of 0.15 M NaCl solution and 100 *μ*L of 2% rabbit erythrocytes. All results were observed macroscopically and the tests were done in triplicate.

A standard haemagglutinating inhibition test was also performed to certify that the interaction of CGN and AFAL does not occur through carbohydrate recognition domain (CRD). AFAL dissolved in 0.15 M NaCl (2 mg/mL) was used. The AFAL samples were diluted in 0.15 M NaCl to give a concentration corresponding to 4 hemagglutinating units (HU) activity per mL. In this study, the dilution was made only to the second tube and then the titer was calculated as 2^2^ (the highest dilution of the AFAL that can agglutinate erythrocytes, that is, the titre, defined as containing one hemagglutinating unit per milliliter). In assay tubes, the carrageenan solution (5 mg/mL in 0.15 M NaCl) was mixed with AFAL solution and incubated at 37°C for 30 minutes. The assay tubes were then left at room temperature for 1 h before 0.2 mL of 2% rabbit erythrocytes were added to each tube. The HA with and without addition of CGN at the same time was tested. The results were observed macroscopically and were expressed as number of hemagglutinating units (HU.100 *μ*L^−1^) which is calculated from the inverse of the highest dilution titer still having visible agglutination.

### 2.2. Primary Structure Analysis of *Acacia farnesiana* Lectin-Like Protein

The BLAST program [26] was used for a comparative analysis of the amino acid sequence of AFAL with the amino acid sequence of other proteins. The best sequences with resolved three-dimensional structures were chosen for alignment. The alignment was performed with Protein Data Bank (PDB) deposited protein structures: 1FAT, 1LUL, 3USU, 2SBA, 1FNY, 2BQP, 1UZY, and 3N35. The multiple alignment with selected lectins was done using Multalin tool [27].

### 2.3. Molecular Modeling by Homology of *Acacia farnesiana* Lectin-Like Protein

Models were obtained from known structures of proteins that have the highest degree of identity among the primary structures aligned with AFAL. The structures were created from the homology molecular modeling server SWISS-MODEL [28], a free online program of Swiss Institute of Bioinformatics. This program allows a fully automated protein structure homology modeling, accessible via the ExPASy web server. Structures from *Phaseolus vulgaris* lectin (1FAT), *Dolichos biflorus *lectin (1LUL), *Butea monosperma *lectin (3USU), *Glycine max *lectin (2SBA), *Robinia pseudoacacia *lectin (1FNY), *Pisum sativum *lectin (2BQP), *Erythrina crista-galli* lectin (1UZY), and *Erythrina corallodendron* lectin (3N35) were compared in order to obtain the high homology.

### 2.4. Molecular Docking

Molecular docking was performed with Hex 6.3 molecular graphics program [29]. The Hex is a fast molecular docking program for calculating and displaying interactions and feasible docking modes of pairs of protein and DNA molecules. It can superpose pairs of molecules using only knowledge of their 3D shapes and calculate protein-ligand interactions using spherical polar Fourier (SPF) correlations to accelerate the docking calculations.

The structural model of AFAL obtained from molecular modeling was used to test the binding to different carbohydrate compounds and to estimate possible binding sites of the sugar and other ligands. This was done in order to explain the biological activities provided by this protein.

An oligosaccharide obtained from the structure of the *Lathyrus ochrus* lectin (deposited under PDB code 1LGC) was used [30]. This oligosaccharide with 2.1 kDa was used because it is bound to a lectin structure which has a relative high identity with AFAL of 40%.

The Iota-carrageenan obtained under the PDB code 1CAR [31] and another oligosaccharide molecule assembled with nine residues of *N*-acetylglucosamine (GlcNac) were used as ligands for molecular docking. These last ligand coordinates were generated using the program PRODRG Server [32]. The (GlcNac) oligosaccharides used in inhibition of hemagglutination test showed positive results only above eight residues. The complexes were named AFAL_glycan, AFAL_CNG, and AFAL_GlcNAc. The molecular docking for two AFAL monomers was performed to obtain the AFAL dimer form.

### 2.5. Biological Activity

All protocols were approved by the USP Ethics Committee (USP, N° 183-2011). Mice received intraperitoneal (i.p.) CGN (500 *μ*g/cav.) dissolved in 0.5 mL of sterile saline. AFAL (1 mg/Kg) was administered intravenously (i.v.) in 0.1 mL of saline 15 minutes before the CGN injection. As a negative control, an experiment was performed in mice that received saline i.v.

The neutrophil migration was evaluated 4 hours after injection of CGN. To this end, the animals were sacrificed by cervical dislocation. Then the cells in the peritoneal cavity were collected by rinsing with 3.0 mL of saline containing 5 IU/mL of heparin. The abdomens of the animals were gently massaged, and through an incision peritoneal fluids were collected.

The total and differential counting of leukocytes was performed according to methodology previously described by De Souza and Ferreira [33]. In this procedure, 20 *μ*L of fluid collected from each animal was diluted in 380 *μ*L of Turk reagent and subsequently used for total leukocyte count in a Neubauer chamber. The differential cell count was performed using stained smears on slides, 50 *μ*L of exudate were centrifuged in cytocentrifuge at 400 ×g for 10 minutes; after this process the smears were stained with hematoxylin-eosin (HE) and the cells counted using an optical microscope and the results expressed as the mean ± standard error mean (SEM) number of cells ×10/mL of peritoneal fluid.

For the assessment of vascular permeability, the mice received AFAL at a dose of 1 mg/kg i.v. 15 minutes before administration of the stimulus CGN (500 *μ*g/cav.). Control group received only saline i.p. One hour before the sacrifice, the animals received Evans blue (50 mg/kg) in plexus eye [34]. The animals were sacrificed by cervical dislocation 3 hours after administration of CGN, and then the fluid in the peritoneal cavity was collected by rinsing injecting 3.0 mL of saline containing heparin (5 UI/mL). The abdomens of the animals were gently massaged and an incision was made. Then, it was collected about 1.0 mL of peritoneal fluid. To quantify the Evans blue extravasation into the peritoneal cavity, spectrophotometry was carried out by reading the optical densities using a wavelength of 610 nm. The absorbance data obtained were converted to mg of Evans Blue, by linear regression based on a standard curve of Evans blue. Results were calculated as mg of Evans blue/mL of peritoneal fluid and expressed as mean ± SEM.

## 3. Results

### 3.1. Hemagglutinating Activity of AFAL

The haemagglutination activity (HA) was observed after 24 hours with a titer calculated as 4 HU.100 *μ*L^−1^. The inhibition of haemagglutination activity of AFAL by CGN had not been effective and it was observed haemagglutinating activity against the rabbit erythrocytes in the first hour after incubation with the same titer previously obtained in the haemagglutinating test. The test showed that the CGN was not capable of inhibiting the binding of protein to erythrocyte membrane carbohydrates suggesting that CGN binding does not occur in the known carbohydrate binding site of legume lectins.

### 3.2. AFAL Model Structure

**Figure 1:**
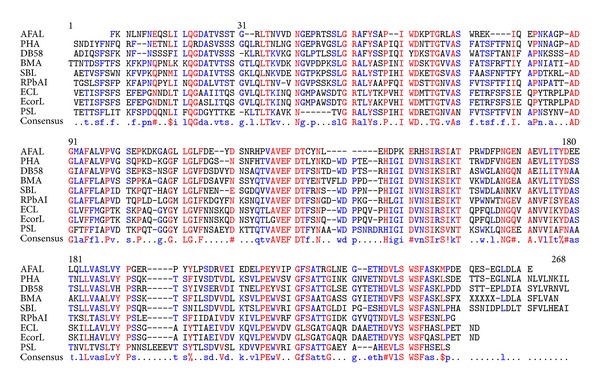
Alignment of amino acid sequences of the AFAL with some lectins. Lectins of *Phaseolus vulgaris* (PHA), *Dolichos biflorus* (DB58), *Butea monosperma* (BMA), *Glycine max* (SBL), *Robinia pseudoacacia* (RPbAI), *Erythrina crista-galli* (ECL), *Erythrina corallodendron* (EcorL) and *Pisum sativum* (PSL).

*Phaseolus vulgaris* agglutinin (PHA) showed high max score in the BLAST alignment, but other proteins present also acceptable identities as can be seen in the multiple alignment in Figure 1. The AFAL sequence analysis showed the highest identity among plant lectins with PHA (64%). However, the molecular modeling of AFAL structure does not show favorable results when the model chosen was PHA, with the *Z*-score of −3.7 (Table 1). Low quality model is expected to have strongly negative *Z*-scores (−1 to −4) featuring the model as unworkable. This apparent discrepancy occurs because the ideal template has high sequence identity, secondary structure similarity, and structural domain assignment.

New templates with favorable result of the BLAST alignment were tested. Lectins of *Dolichos biflorus* (58%), *Butea monosperma* (51%), *Glycine max* (50%), *Robinia pseudoacacia* (48%), *Pisum sativum* (42%), *Erythrina crista-galli* (38%), and *Erythrina corallodendron* (37%) were identified as the best ones.

All similar proteins were used as templates for molecular modeling by SWISS-MODEL program, but only the chain A of *Pisum sativum* lectin (PSL) (PDB code 2BQP) [35] showed high *Z*-score which converges to a statistically valid structural model (Table 1). Dimeric PSL is also a mannose/glucose specific metalloprotein and its structure provided the best modeling template at 1.90 Å resolution with sequence identity of 42% [36].

**Figure 2:**
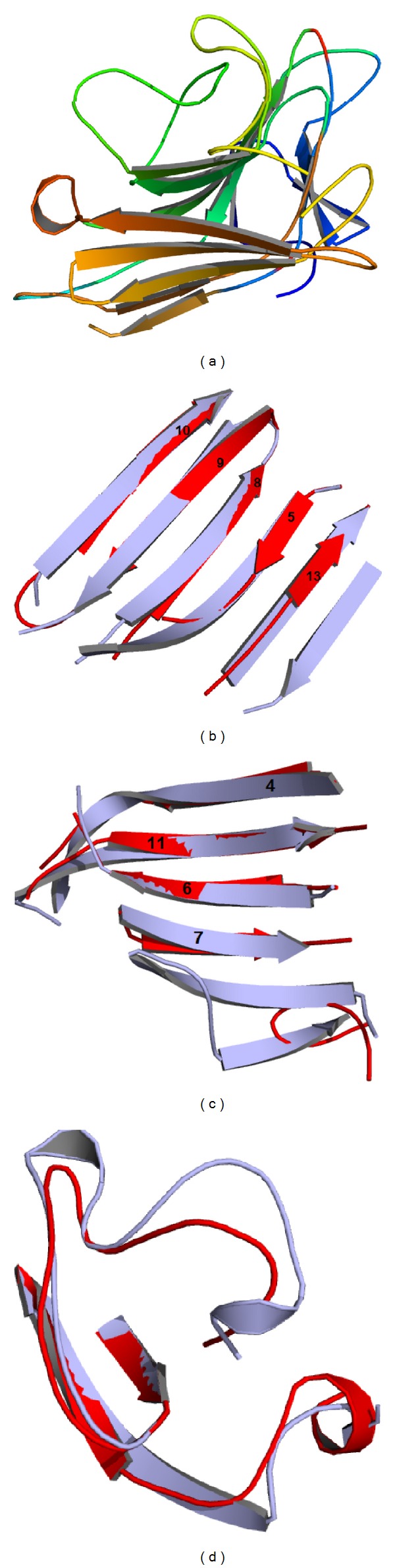
AFAL model obtained by molecular modeling. (a) *β* sheets domains: posterior, frontal, and side. (b) Superposition of PSL posterior domain in light gray and AFAL posterior domain in gray. The *β* strands are numbered according to the AFAL model topology. (c) Superposition of PSL frontal domain in light gray and AFAL frontal domain in gray. (d) Superposition carbohydrate binding site of PSL in light gray and AFAL carbohydrate binding site in gray.

The AFAL structure model is folded as a *β* sandwich (thirteen *β* strands), as previously observed in legume lectins. The monomer model shows three *β* sheets domains: a frontal one, a posterior one, and another positioned at the side (Figure 2(a)). The model differs from the PSL template, in loops regions, number of *β* strands (sixteen in PSL), and carbohydrate binding site. The AFAL model structure contains five *β* strands in the posterior *β* sheet domain (Figure 2(b)). The common canonical oligomeric structures in legume lectins show six *β* strands. In addition, two *β* stands are missing in the frontal *β* sheets domain of the AFAL model (Figure 2(c)).

The formation of dimer canonical structures is common in legume lectins, but the absence of a *β* strand in canonical domain (posterior *β* sheets) prevents the possible formation of the conventional canonical dimer in AFAL model. The absence of the two helixes in the AFAL structure (Figure 2(d)) alters the carbohydrate binding site folding probably not allowing the binding to simple carbohydrates.

### 3.3. Molecular Docking

**Figure 3:**
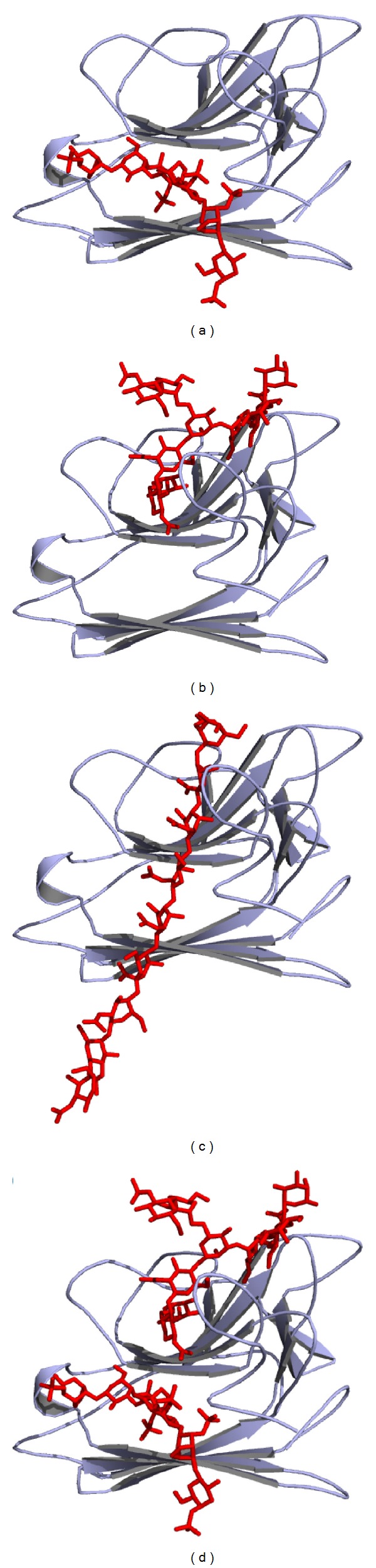
AFAL_molecular docking. (a) The AFAL_molecular docking with carrageenan. (b) The AFAL_molecular docking with oligosaccharide. (c) The AFAL_molecular docking with *N*-acetylglucosamine (GlcNac). (d) AFAL binding the carrageenan and oligosaccharide in different sites.

Structural superposition of AFAL/PSL showed two major differences. First, AFAL has an absent *β* strand in the posterior *β* sheet domain and two absent in frontal *β* sheet domain, conferring AFAL a differentiated characteristic from other legume lectins: its possible ability to bind to CGN. This is confirmed by molecular docking of AFAL_CGN that shows negative E-value for interactions. The presence of the loop in the space that was occupied by two *β* strands in other legume lectins allows the binding of the CGN molecule (Figure 3(a)). Second, AFAL has an absence of a helix in carbohydrate binding site region, which prevents the binding to monosaccharides.

AFAL molecular docking, performed with CGN, (Figure 3(a)) revealed that CGN interacts with amino acids Ser129, Ala131, Leu156, Ser172, Asp173, Arg174, Ile130, Glu180, and Ala108 through hydrogen bonds, as described in Table 2. AFAL_CGN interactions show favorable binding energy with *E*-value −328.430 Kcal·mol^−1^.

AFAL glycan molecular docking also shows negative *E*-value confirming the integrity of the carbohydrate binding recognition domain (Figure 3(b)). This showed that both bind to AFAL on different sites (Figure 3(d)). The AFAL glycan molecular docking was performed with the aim of confirming that the CGN was not able to inhibit the AFAL hemagglutination activity. AFL_glycan interactions show favorable binding energy with *E*-value −445.4719 Kcal·mol^−1^. The AFAL_glycan interaction occurred through amino acids Asp73, Gly92, Leu93, Leu94, Leu96, Phe97, Glu110, Cys114, Asn116, Glu118, His119, Asp120, Gly194, Leu195, Glu197, and Asn512 establishing 34 hydrogen bonds.

Polymers of up to eight residues of *N*-acetylglucosamine (GlcNac) were ineffective to inhibit HA. Although, the AFAL_GlcNac oligosaccharide molecular docking revealed interactions that show favorable binding energy with *E*-value −313.5632 Kcal·mol^−1^. The AFAL_GlcNac oligosaccharide interaction (Figure 3(c)) occurred through amino acids Gly92, Leu93, Glu110, Asp112, Asn116, Glu118, His119, Ser129, Ile 130, Asp173, and Ser172 establishing 21 hydrogen bonds. The best result for AFAL monomers interactions shows favorable binding energy to dimer formation with *E*-value −518.6367 Kcal·mol^−1^.

### 3.4. Biological Activity

**Figure 4:**
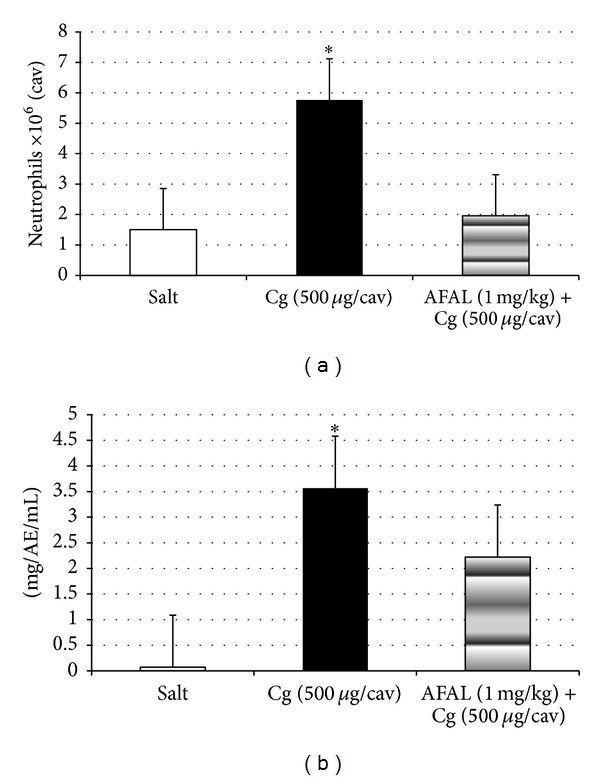
Antiinflammatory activity of AFAL. (a) Anti-inflammatory effect of *A. farnesiana* on carrageenan-induced peritonitis in mice. Mice were treated with saline (0.1 mL, i.v.) or AFAL (1 mg/kg, i.v., 15 minutes before) and then injected i.p. with carrageenan at a dose of 500 *μ*g/cavity. The neutrophil migration was evaluated 4 h later. The white bars represent the neutrophil migration induced by saline injected i.p. The values are means ± SD. *P < 0.05 compared to carrageenan group. (b) AFAL reduces vascular permeability on carrageenan-induced peritonitis in mice. Effects of the pretreatment with AFAL on vascular permeability. Vehicle (saline) or AFAL (1 mg/kg) was injected i.v. and, 15 minutes later, carrageenan (500 *μ*g/cavity) was injected. The values are means ± SD. *P < 0.05 compared to carrageenan group.

The i.v. administration of AFAL, 15 minutes before stimulation by CGN, reduced the intense migration of neutrophils into the peritoneal cavity at a dose of 1 mg/kg four hours after CGN i.p. administration (500 *μ*g/cav.). This inhibition was 71% and the positive control group (CGN) produced a significant increase in neutrophils as compared to saline group (Figure 4(a)).

The CGN i.p. administration (500 *μ*g/cav.) induced a significant increase in vascular permeability in the control group, seen through the leakage of dye (Evans blue) to the intraperitoneal fluid. The pretreatment of the animals with intravenous AFAL (1 mg/kg) reduced significantly the alteration of vascular permeability (40% inhibition) in the cavity induced by CGN (Figure 4(b)). So, even with a small reduction of the permeability the anti-inflammatory effect is very efficient in reducing the number of cells in the peritoneum.

## 4. Discussion

Santi-Gadelha and coworkers [1] characterized AFAL as PHA-like protein based on their amino acid sequence and biochemical characteristics. Among these characteristics, the inability to bind to simple carbohydrates, to chitin-like oligosaccharides (two to eight residues GlcNAc), and to ovomucoid glycoprotein (rich in GlcNAc) was the most intriguing and determined the need to study the possible interactions with complex carbohydrates based on docking simulations. Docking with complex glycan, GlcNac oligosaccharide and CGN are corroborated by previous results of haemagglutinating activity and by the purification protocol of AFAL using chitin affinity chromatography column [1] and also by the HA of this work.

AFAL structural analysis showed that CGN and glycan binding domains are independent. This is in accordance with our findings for the inhibition test (HA) by CGN, in which no change was observed in agglutination after incubation, probably due to the fact that carbohydrate binding site is not affected by the presence of CGN that binds in another site. It is also an important observation that the presence of CGN bound with AFAL improves its capacity to agglutinate cells. The HA in this condition was observed only after the first hour, but without CGN the observation was made only after twelve hours.

The ability to AFAL binding CGN can be explained by absence of the two *β* stands in frontal *β* sheets and the sixth *β* strand in posterior region seems to be responsible for its inability to form stable dimers. The amino acid composition analysis of the fifth *β* strand of AFAL differs from PSL lectin by presence of Ala209-His230, Lys211-Glu232, and Phe208-Ser229. The canonical dimers are formed by interface interactions between the amino acid side chains by a large stranded *β* sheet resulting from the association of the two 6-stranded back sheets [14, 37] and the differences in these interactions result in lack of dimer stability.

This change in the side chains causes a structural alteration which means the deletion of a posterior *β* strand on AFAL and increases loop region also preventing the stabilization and formation of the dimer. The absence of two strands in the frontal *β* sheet allows AFAL to bind to CGN. Structurally, the presence of these particular *β* strands in other legume lectins structures [14, 37] does not allow the stabilization and binding of CGN.

**Figure 5:**
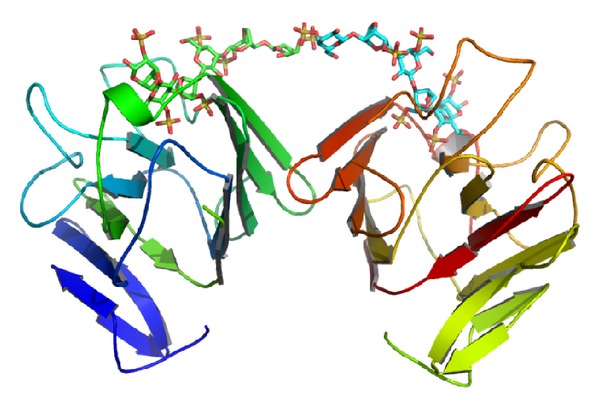
The connections between the monomers are stabilized by CGN. This interaction contributes to the stability of the tetramer.

The time-dependent oligomerization states of the AFAL, observed by Santi-Gadelha and coworkers [1], could be stabilized in the presence of CGN, reducing the hemagglutination time of twelve hours to the one hour. This is due to AFAL_CGN interaction that positively contributes to the formation of the dimer (Figure 5). This small increase in stability can allow the formation of fine AFAL crystals and possibility that its structure could be resolved by X-ray crystallography (data not shown) as occurred in other lectins when bound to ligands (e.g., *Parkia platycephala* lectin—PPLI) [38].

Probably, the difficulty in obtaining AFAL crystals is related to the heterogeneity of samples that is a result of pools of proteins in different states of oligomerization that do not stabilize as tetramers (this was called time-dependent oligomerization), which is mainly caused by the reduced energy needed to disrupt the tetramer compared to other legume lectins.

Therefore, these features should represent a significant difference about the anti-inflammatory mechanism presented by AFAL from those presented by other legume lectins. It has also been shown that legume lectins can be proinflammatory when administrated subcutaneously or anti-inflammatory when this administration is intravenous [20]. Alencar and coworkers [39] demonstrated that legume lectins act in inflammatory process via carbohydrate recognition domain (CDR), but AFAL is also capable of acting in a different way based on its affinity by CGN. AFAL was previously characterized as proinflammatory by Santi-Gadelha through paw edema model (s.c.) [40]; this present study shows that in complex with CGN the action is completely different, being significantly anti-inflammatory. The biological activity assay of AFAL showed that intravenous administration of this protein reduced neutrophil migration into the peritonea cavity and vascular permeability induced by CGN, processes that are related to carbohydrate recognition. From these data, it is possible to make a relationship between anti-inflammatory activity of AFAL and CGN. This sulfated polysaccharide has been used to test inflammation and anti-inflammatory activity with lectins for long time, but little can be deduced about how this activity occurs [41–43]. Some authors assumed that some anti-inflammatory events, such as inhibition of neutrophil migration, occur through competition for binding to neutrophils between lectins tested and selectins [20, 39], but AFAL also can inhibit the signaling pathway triggered by CGN through the recognition and binding, preventing the recognition of carrageenan by epithelial cells and inhibiting the induction of IL-8 production resulted from the activation of BCL10 [23, 25].

It can be observed that the main reduction caused by AFAL was the neutrophil migration instead of the vascular permeability. This could be due to the chemotaxis, mainly caused by the increase in IL-8 levels induced by carrageenan, which reduces the adhesion and rolling of neutrophils [23, 44].

Thus, we postulated that the AFAL differs from other legume lectins in anti-inflammatory activity by structural absence of *β*-strands which allows direct connection to CGN and at the same time with glycans, decreasing the amount of CGN that interacts with cells and reducing the inflammation effects. The presence of two independent binding sites in AFAL allows the enhancement of the defense mechanism of the organism and simultaneously preserves its activity of binding to carbohydrates as demonstrated by inhibition with carbohydrates, in which HA was not reversed by addition of CGN. This way, AFAL probably has the capacity to interfere in the inflammatory process through the reduction of IL-8 and simultaneously exert its activity of binding to carbohydrates as demonstrated by inhibition tests and could also compete with selectins.

## Figures and Tables

**Table 1:** Templates for molecular modeling by SWISS-MODEL program.

Code	Template	Score	Identity %
1FAT	*Phaseolus vulgaris* lectin	−3.7	64
1LUL	*Dolichos biflorus *lectin	−3.99	58
3USU	*Butea monosperma * lectin	−3.18	51
2SBA	*Glycine max *lectin	−3.32	50
1FNY	*Robinia pseudoacacia * lectin	−3.75	48
2BQP	*Pisum sativum * lectin	0.00	42
3N35	*Erythrina corallodendron * lectin	−3.86	38
1UZY	*Erythrina crystagalli * lectin	−3.87	37

**Table 2:** The overall interactions between AFAL and carrageenan.

CARRAGEENAN	Ser129		Ala131		Leu156	Ser172	Asp173		Arg174	Ile130	Glu180	Ala108
	O	OG	O	N	O	O	OD2	O	O	N	OE2	O
G4S3^1^												
O1	2.8	—	—	—	—	—	—	—	—	3.2	—	—
O2	3.2	—	—	—	—	—	—	—	—	—	—	—
G4S5												
O2	—	—	—	—	—	—	—	—	3.1	—	—	—
O6	—	—	—	—	—	—	—	—	—	—	3.3	—
O7	—	—	—	—	—	—	—	—	—	—	—	3.2
DGS2^2^												
O7	—	—	2.5	2.3	—	—	—	—	—	—	—	—
O9	—	—	—	3.2	—	—	—	—	—	—	—	—
DGS4												
O3	3.3	3.0	—	—	—	—	—	—	—	—	—	—
O5	—	—	—	—	—	—	3.1	—	—	—	—	—
O7	—	—	—	—	2.8	3.2	—	2.5	—	—	—	—
O8	—	3.3	—	—	2.9	—	—	—	—	—	—	—

^1^G4S: 4-O-sulfo-*β*-D-galactopyranose; ^2^DGS: 3,6-anhydro-D-galactose-2-sulfate.

## Abbreviations

AFAL: *Acacia farnesiana* lectin-like protein
CGN: Carrageenan
CRD: Carbohydrate recognition domain
GlcNac: *N*-acetylglucosamine
HA: Haemagglutinating activity
HU: Hemagglutinating units
PHA: *Phaseolus vulgaris* agglutinin.
